# Molecular Cloning and Bioinformatics Analysis of *DQA* Gene from Mink (*Neovison vison*)

**DOI:** 10.3390/ijms20051037

**Published:** 2019-02-27

**Authors:** Zhaobin Fan, Houfeng Zhang, Min Rong, Dongmei Meng, Zhenxing Yu, Lili Jiang, Peihong Jiang

**Affiliations:** 1 College of Pharmacy, Heze University, Heze 274015, China; fanzhaobin08@163.com (Z.F.); 13953025825@163.com (H.Z.); dongmeimeng@163.com (D.M.); yuzhenxing10@163.com (Z.Y.); 2 Institute of Specialty Animal and Plant Sciences of CAAS, Chinese Academy of Agriculture Science, Changchun 130112, China; rongmin12@163.com

**Keywords:** major histocompatibility complex, mink, *DQA* gene, *Neovison vison*

## Abstract

In the present study, we cloned, sequenced, and explored the structural and functional characteristics of the major histocompatibility complex (MHC)-*DQA* gene from mink (*Neovison vison*) for the first time. The full-length sequence of *DQA* gene was 1147-bp-long, contained a coding region of 768-bp, which was predicted to encoding 255 amino acid residues. The comparison between *DQA* from mink (*Neovison vison*) and other *MHC-DQA* molecules from different animal species showed that nucleotide and encoded amino acid sequences of the mink *DQA* gene exhibited high similarity with the ferret (*Mustela pulourius furo*). Phylogenetic analysis revealed that mink (*Neovison vison*) *DQA* is grouped with that of ferret (*Mustela pulourius furo*). The cloned sequence contained a 23-amino acid NH_2_-terminal signal sequence with the signal peptide cutting site located in amino acids 23–24, and had three Asn-Xaa-Ser/Thr sequons. Three cysteine residues were also identified (Cys-85, Cys-121, and Cys-138). The 218 to 240 amino acids were predicted to be the transmembrane domains. The prediction of the secondary structure revealed three α-helixes and fourteen β-sheets in *Neovison vison DQA* protein, while random coil was a major pattern. In this study, the whole CDS sequence of *Neovison vison DQA* gene was successfully cloned, which was valuable for exploring the function and antiviral molecular mechanisms underlying the molecule. The findings of the present study have laid the foundation for the disease resistance and breeding of mink.

## 1. Introduction

The major histocompatibility complex (MHC) is a chromosomal region consisting of a group of closely linked loci. It binds to specific epitopes, which is the key to triggering the immune system to attack the virus-infected cell through the proteins involved in the immune response of the body [1,2,3]. Due to the polygenic characteristics, MHC was divided into MHC class I, II, and III genes according to the differences in the structure, tissue distribution, and function of the coding molecules [4]. The class II genes of MHC encoded for α and β chains of DR and DQ dimer molecules, which present antigenic peptides to the helper T cells and was considered as a candidate gene for animal disease-resistant breeding [5,6,7]. A striking characteristic of *MHC II-DQA* genes is their extreme polymorphism [8,9,10]. Due to the central role of *DQA* gene in the vertebrate immune system, it is presumed that one of the main selective pressures promoting MHC diversity gives rise to the susceptibility or resistance against infectious diseases and autoimmune disorders [11,12].

Presently, with the development of the mink industry in China, the number of mink raised reaches is ~33 million [13]; however, various diseases have become the obstacle to the further rapid development of the mink breeding industry, which mainly includes hemorrhagic pneumonia and Aleutian Mink Disease (AMD), especially the latter [14,15]. AMD is caused by the Aleutian Mink Virus (ADV) and is combined with high mortality, reduced pregnancy rates, decreased litter size, and abortion, which results in severe economic loss in mink farming globally [16]. Vaccination is not feasible because of the peculiar pathogenic mechanism of AMDV, and the only possible method to be rid of this virus is to implement eradication measures, which consist of identifying and culling the infected animals [17]. Nevertheless, such an approach is time- and energy-consuming as well as cost-inefficient. Therefore, gene screening to improve the disease resistance of mink is crucial. The polymorphism of *DQA* molecules appear to be responsible for variations in the immune responses of individuals to antigens, which in turn might contribute to resistance against autoimmune diseases. Therefore, *DQA* can be considered a candidate gene for mink disease-resistant breeding.

Presently, only a few studies have focused on the mink in the *MHC II* gene worldwide. Cong et al. cloned the *MHC II DRA* genes of *Neovison vison* [18]. Becker et al. cloned the partial exon 2 of the *DRB* gene of European mink and obtained different isoforms [19]. Fan et al. cloned the full-length sequence of the *DQB1* gene and analyzed the polymorphism of exon 2 in five mink breeds [20]. Hitherto, none of the studies have reported the cloning of the *DQA* gene from mink with rich polymorphism and correlated it to disease resistance. In the present study, the full-length sequence of *DQA* gene form mink was cloned and sequenced for the first time, and the bioinformatics characteristics were analyzed. Thus, this study would provide a deep insight into the disease control in mink as well as the MHC diversity in common animals to investigate the immunological functions, and selective and evolutionary forces that affect the MHC variation within and among the species.

## 2. Results

### 2.1. Molecular Cloning of DQA from Neovison vison

**Figure 1:**
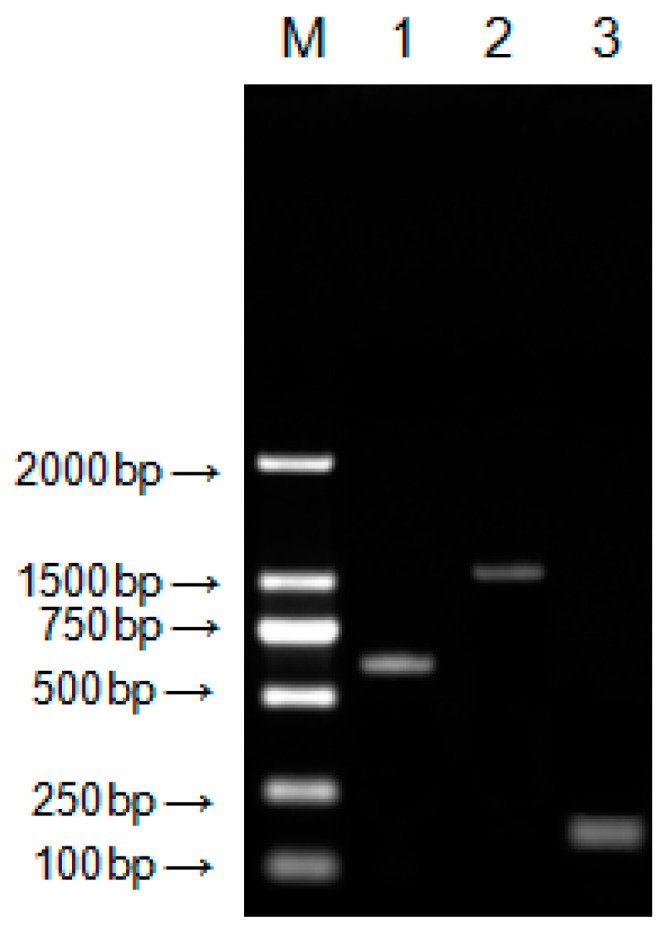
Agarose gel electrophoresis of Neovison vison *DQA* gene amplification. M: DL2000 DNA marker; (1) PCR product with primer pair F1U/F1L; (2) PCR Product with primer pair F2U/F2L; and (3) PCR product with primer pair F3U/F3L.

**Figure 2:**
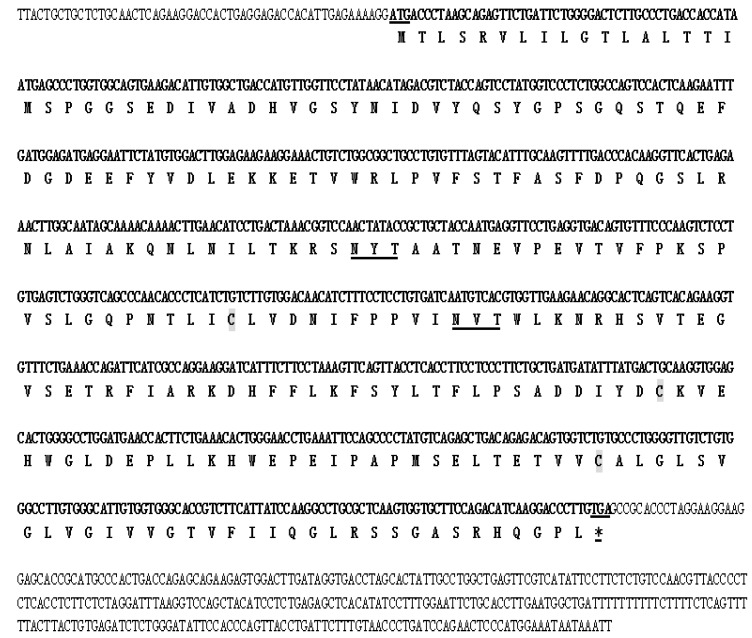
The nucleotide and deduced amino acid sequences of *Neovison vison DQA*. The deduced amino acid sequence is reported in one-letter code. The start is in boldface type, and the underlined and stop codon is showed with asterisks. The three cysteine residues (Cys-133, Cys-189, and Cys-221) are highlighted in gray. Two potential Asn-Xaa-Ser/Thr sequons were also observed in the sequence (underlined in boldface type).

The NCBI DQA sequence of *Mustela putorius furo* and *Enhydra lutris kenyoni* were used for molecular cloning of *DQA* from *Neovison vison*. The amplified cDNA was separated by electrophoresis on 1.5% agarose gel, which showed the expected band size as compared to the standard molecular weight ladder (Figure 1). This fragment was excised from the agarose gel, purified, ligated in the TA vector pMD19-T (TaKaRa Biotechnology Co., Ltd., Dalian, China), and cloned in *E. coli*. The positive clones were confirmed by colony PCR. The amplicons were obtained by PCR amplification of the cDNA with gene-specific primers encoding the *DQA* gene (Figure 1). The amplicons were purified, cloned, and characterized by sequencing. This sequence represented the first cloned *DQA* from *Neovison vison*, which encompasses the full coding region in comparison with the corresponding regions from different organisms. Sequence analysis revealed that the cDNA encoding the *DQA* gene consisted of 1147-bp open reading frame (ORF) with a 768-bp encoding a 255-aa polypeptide of 28 kDa molecular mass (Figure 2). The predicted protein sequence of *Neovison vison DQA* by EditSeq (DNAStar) revealed the presence of 19 strongly basic (+) (K, R), 29 strongly acidic (−) (D, E), 95 hydrophobic (A, L, F, W, and V), and 69 polar amino acids (N, C, Q, S, T, and Y) with isoelectric point at 4.965 and −9.125 charge at pH 7.0. The gene sequence comprised of 24.09% A (185), 24.61% G (189), 26.56% T (204), 24.74% C (190), 50.65% A+T (389), and 49.35% C+G (379). 

The sequence analysis for the coding region of *Neovison vison DQA* showed a high similarity (86.6–97.5%) with *DQA* from other species: 97.5% ferret (*Mustela pulourius furo*), 94.0% sea otters (*Enhydra lutris*), 92.2% sea bear (*Ursus maritimus*), 91.9% giant panda (*Ailuropoda melanoleuca*), 90.8% walrus (*Odobenus rosmarus divergens*), 90.5% Sea lions (*Zalophus*), 90.5% Hawaiian monk seal (*Neomonachus schauinslandi*), 89.6% wolf (*Canis lupus*), and 86.6% horse (*Equus caballus*). The deduced amino acid sequences showed a maximum divergence of 23.3%, with sequence identities ranging from 80.0 to 96.9% as compared to other species. Furthermore, DQA proteins shared 96.9% and 89.4% identity with ferret (*Mustela pulourius furo*) and sea otters (*Enhydra lutris*), respectively. Also, 88.2% identity was detected with sea bear (*Ursus maritimus*) and giant panda (*Ailuropoda melanoleuca*), 86.7% with walrus (*Odobenus rosmarus divergens*), 86.3% with sea lions (*Zalophus*) *and* wolf (*Canis lupus*), 85.1% with Hawaiian monk seal (*Neomonachus schauinslandi*), and 80.0% with horse (*Equus caballus*) proteins. 

**Figure 3:**
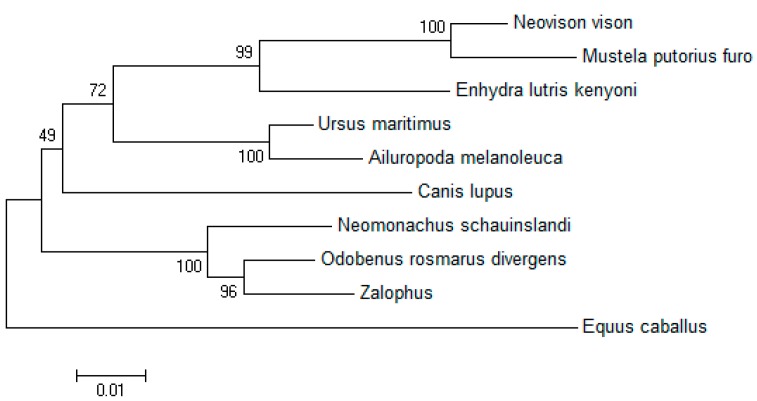
*DQA* genetic phylogenetic tree. The phylogenetic tree was constructed using the neighbor-joining method. The results were confirmed by bootstrapping values at the nodes, which indicate bootstrap percentages for the given split among 1000 repetitions.

Both sequences were aligned with the retrieved sequences using Clustal (W) method from Mega 5.2 software, and percent identity and divergence were determined. Several clades were formed during the phylogenetic analysis of the broad class of *DQA* gene. *Neovison vison* and *Mustela pulourius furo* formed one monophyletic clade, whereas that from other species except *Equus caballus* formed a separate clade. The *Neomonachus schauinslandi DQA* was grouped closely with that of *Odobenus rosmarus divergens* and Zalophus (Figure 3).

### 2.2. Molecular Characterization of DQA from Neovison vison

#### 2.2.1. Hydrophilic and Hydrophobic Analysis of *Neovison vison* DQA Protein

**Figure 4:**
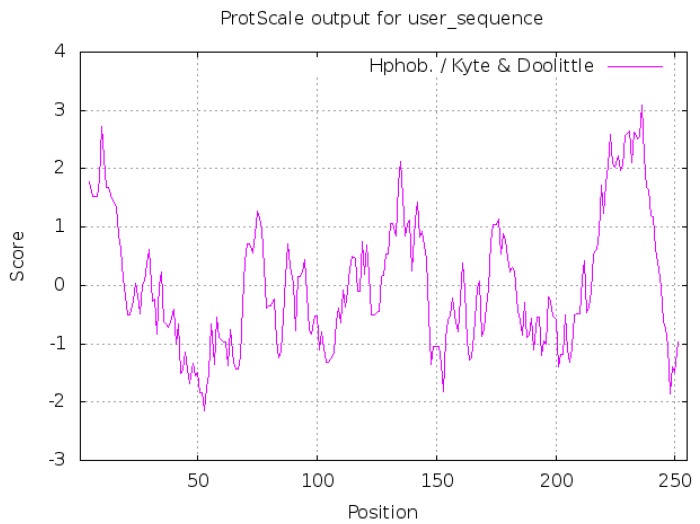
The hydrophobicity profile of *Neovison vison* DQA protein. Y-axis displays the hydrophilic index: a positive number indicates hydrophobicity, the greater the value, the greater the hydrophobicity; negative numbers indicate hydrophilicity, smaller values indicate stronger hydrophilicity. The x-axis displays the position of DQA amino acids.

ProtScale was used to analyze the hydrophilicity and hydrophobicity of DQA protein, and Hphob./Kyte & Doolittle were selected as the prediction standards. Figure 4 presented three distinct hydrophobic regions, with the highest score 3.089 at the position 236Thr (T) of the polypeptide chain, which represented the strongest hydrophobicity, while the lowest score −2.144, at position 53Asp (D), represented maximal hydrophilicity. Since more than half of the amino acid sequences are hydrophobic residues, the entire polypeptide chain is deemed as such with an average hydrophobic index of 0.300.

#### 2.2.2. Prediction of Transmembrane Helical Structure of *Neovison vison* DQA Protein

**Figure 5:**
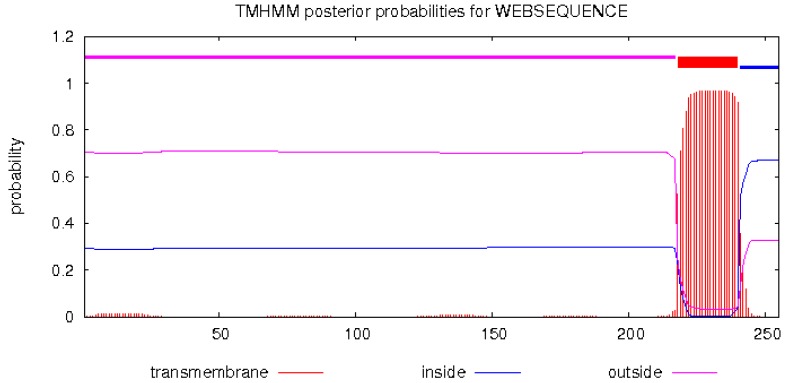
Prediction of transmembrane region of *Neovison vison* DQA protein.

TMHMM 2.0 analysis tool predicted the transmembrane domain extending from 218–240 amino acids, while 241–255 amino acids are in the cells, and 1–217 amino acids are outside the cells (Figure 5). This result was in agreement with the predicted hydrophobic peak (Figure 4), which further verifies that this area might be a transmembrane region.

#### 2.2.3. Prediction of Signal Peptide of *Neovison vison* DQA Protein

**Figure 6:**
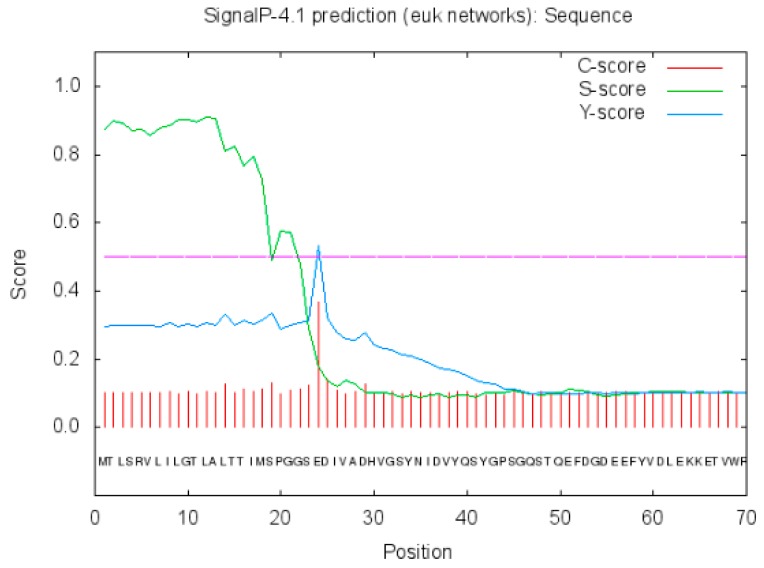
Prediction of signal peptide of *Neovison vison* DQA protein.

SignalP 4.1 Server predicted the signal peptide sequence of 23 amino acids in the protein (Figure 6 for the signal peptide prediction).

#### 2.2.4. Modification Prediction of the *Neovison vison* DQA Protein Structure

**Figure 7:**
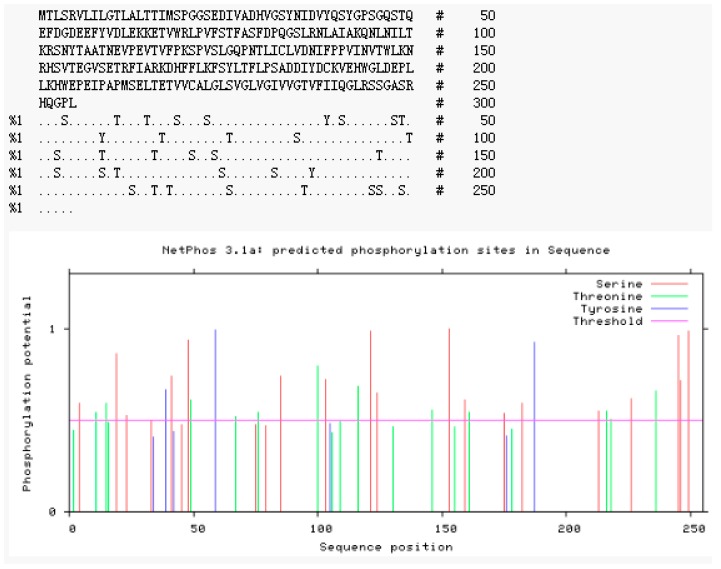
Prediction of phosphorylation sites of *Neovison vison* DQA protein.

**Figure 8:**
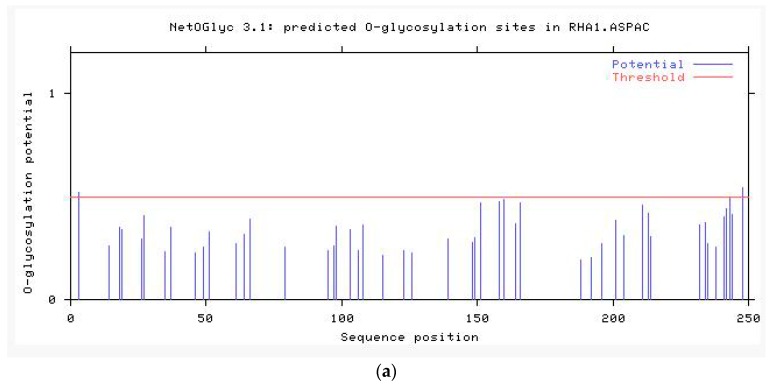

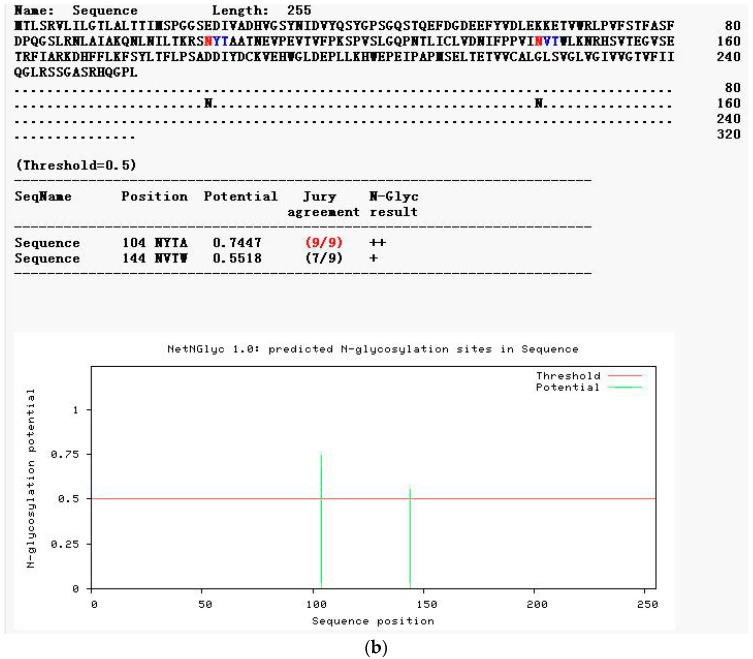
(**a**) Prediction of potential *O*-glycosylation sites of *Neovison vison* DQA protein. (**b**) Prediction of potential *N*-glycosylation sites of *Neovison vison* DQA protein.

NetPhos 3.1 server analysis of the deduced amino acid sequence revealed 34 potent phosphorylation sites, including three threonines (Thr), 18 serines (Ser), and three tyrosines (Tyr) at the threshold of 0.5 (Figure 7). NetOGlyc 4.0 Server and NetNGlyc 1.0 were used to predict the potent *O*-glycosylation and *N*-glycosylation site of DQA, respectively. As shown in Figure 8a, the potent *O*-glycosylation site was predicted at Ser-103 and Ala-106 with probabilities of 0.540611 and 0.536474, respectively. The potent *N*-glycosylation site existed at Thr-104 and Val-144 with probabilities of 0.7447 and 0.5518, respectively (Figure 8b).

#### 2.2.5. Subcellular Localization and Domain Prediction of *Neovison vison* DQA Protein

**Figure 9:**
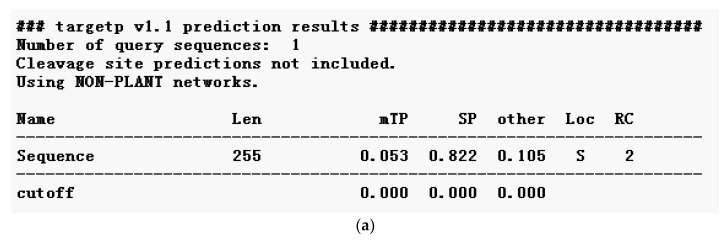

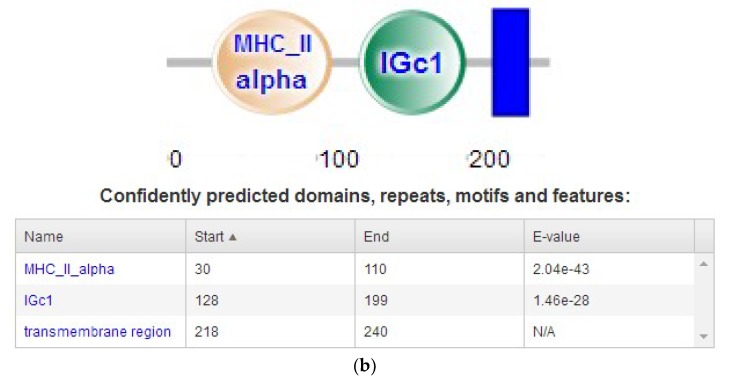
(**a**) The subcellular location analyses of *Neovison vison* DQA. (**b**) Conservative structure domain analysis of *Neovison vison* DQA. Len, Sequence length; Loc, Prediction of localization based on the scores of mTP, SP, and other; RC, Reliability class, scores from high to the end are 1–5; the lower the score, the more reliable the prediction.

The subcellular localization of DQA protein was predicted using Target P1.1 server, which indicated a secretory channel signaling peptide (SP) in the protein (Figure 9a). SMART program was used to predict the structural domain of DQA protein (Figure 9b). Amino acids 30–110 aa are MHC II, 128–199 aa is the IGc1 domain, and 218–240 aa is the transmembrane region (Figure 5). 

#### 2.2.6. Function Prediction of DQA Protein

In this study, ProtFun 2.2 Server analysis predicted the functional domain and classification of the DQA protein. Table 1 lists that the highest probability is voltage-gated ion channel with the probability value 12.682. However, additional tests are required to determine whether it is a Na^+^, K^+^, or Ca^2+^ ion channel.

#### 2.2.7. Advanced Structure Prediction of DQA Protein

**Figure 10:**
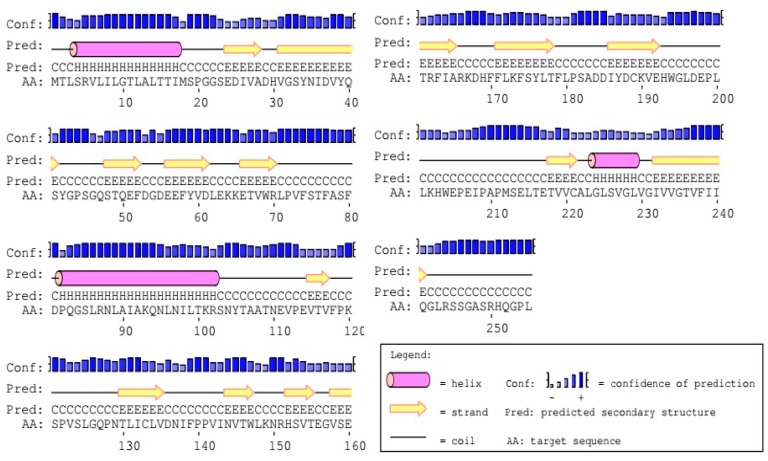
Prediction results of secondary structure of DQA protein.

**Figure 11:**
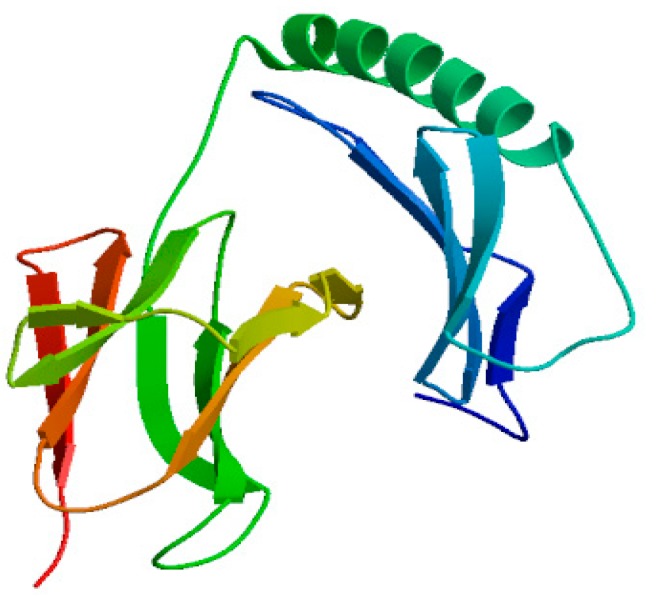
Prediction of the tertiary structure of DQA protein.

The secondary structure of *Neovison vison* DQA using the PredictProtein software predicted that the DQA protein was composed of 19.2% alpha helix, 34.5% beta fold, and 46.3% random coils. Figure 10 displayed the results of PSIPRED analysis, wherein the addition of three alpha helices and fourteen beta folding regions rendered a majority of the amino acids in a state of irregular curls in DQA protein. The homologous modeling of *Neovison vison* DQA protein was carried out using the SWISS-MODEL Server. The tertiary structure of the DQA protein primarily in the form of an alpha helix, beta folding, and random crimp was in agreement with the predicted secondary structure of DQA protein (Figure 11).

## 3. Discussion

The *DQA* gene presents foreign peptide antigens from infectious agents, including bacteria, viruses, and autoimmune antigens [21], providing a valuable source of genetic markers for examining the correlations between host variants and disease resistance or susceptibility [22]. As a functional unit, exon 3 of the swine leukocyte antigen DQA (*SLA-DQA*) gene is pivotal to both diarrhea susceptibility and resistance based on the effects on diarrheal infection in 425 suckling piglets [23,24]. The correlation between the polymorphisms of *SLA-DQA* exon 2 and piglet diarrhea in three Chinese native pig breeds indicated that the amino acid variations in the pepetide binding pockets play a critical role in the antigen-binding groove in piglet diarrhea resistance [25]. Feng et al. demonstrated significant differences in the white blood cell count, red blood cell count, neutrophil ratio, and other immune indicators of GOLA-DQA genotypes, which provide a basis for breeding disease-resistant species of goats [26]. Bao et al. reported that the resistance to *E. coli* F18 infection in weaned piglets is related to the mRNA expression of SLA-DQA [12]. However, whether the DQA gene can be considered as a candidate gene for mink disease-resistant breeding necessitates further researches that focus on the correlation between the polymorphism of DQA gene in mink and susceptibility to disease and disease phenotype.

The phylogenetic tree based on the nucleotide coding sequence of *Neovison vison* DQA gene was divided into three major subgroups as follows. *Neovison vison* had the shortest genetic distance from *Mustela pulourius furo*, followed by *Enhydra lutris*, *Ursus maritimus*, *Ailuropoda melanoleuca*, and *Canis lupus*, and the furthest from *Equus caballus*. The results accorded with the rule of genetic evolution of species, and showed that polymorphism of *DQA* gene in *Neovison vison* harbored cross-species maintenance. The *DQA* gene of mink contained an open reading frame (ORF) of 768 nucleotides, encoding a polypeptide of 255 amino acids, which was similar to ferret, gayal, *Bubalus bubalis*, and Bamei pigs [27,28,29]; however, some differences were observed from that of Wuzhishan pig [30]. Moreover, 19 nucleotide and eight amino acid substitutions were detected in the DQA gene coding regions as compared to the ferret, which indicated relatively abundant polymorphisms. Based on the theories that mutations in different varieties might affect the spatial structure and function of proteins, it can be speculated that *DQA* gene is a potential genetic marker. 

The secretion of proteins depends on the signal peptide located to the N-terminal of the protein precursor. The DQA protein of mink consisted of a signal peptide with 23 amino acid residues, indicating it to be a secreted protein with transmembrane transport function. This phenomenon was consistent with the prediction that DQA protein harbored a transmembrane domain at 218–240 amino acids. It is proposed that the longer the half-life, the more stable is the protein [31]. In this study, the DQA protein was an unstable entity with up to 30 h half-life in mammalian reticulocytes, which might be related to its different physiological functions in different subcellular organelles [32].

*N*-glycosylation is one of the major modifications after translation of eukaryotic proteins, which can affect the antigen-determining clusters, charge properties, and thermal stability of the proteins. The degree of glycosylation and abnormalities in the structure of sugar chains are the markers of cancer, which aid in the early diagnosis and timely treatment of diseases [33,34]. The mink DQA protein consists of two putative *N*-glycosylation sites (N104–Y105–T106 and N144–V145–T146), which are consistent with those in Bamei pigs [29]. The reliability of the predicted glycosylation site of DQA protein in mink and its physiological function needs to be investigated further. In addition, the secondary structure prediction for DQA protein in mink indicated that the percentages of α-helix, β-sheet, and random coil are 19.2%, 34.5%, and 46.3%, respectively. This result was in agreement with that of Bamei pigs, canine, and Yantai black pig [35,36]. The SWISS-MODEL server was used to predict the three-dimensional structure of 25–208 amino acids of mink DQA protein using HLA-DQA1 protein as the homologous modeling tool. The similarity and coverage between mink-DQA and HLA-DQA1 sequences was 78.87% and 76%, respectively. Nevertheless, further exploration of the DQA protein structure would clarify its biological functions and immunological mechanisms.

## 4. Materials and Methods

### 4.1. Experimental Mink

The liver tissue of mink was provided by the Special Animal Genetic Resources Key Laboratory of the Institute of Special Products, Chinese Academy of Agricultural Sciences. 

### 4.2. RNA Extraction, cDNA Synthesis, and Reverse Transcription PCR

Tissue samples (heart, liver, spleen, lung, kidney, intestines, skin, and cureus) were excised, snap-frozen in liquid nitrogen, and stored at −80 °C until further use. Total RNA was extracted from mink tissues using RNAiso Plus (TaKaRa) and quantified by spectrophotometry on the NanoDrop (Thermo Fisher Scientific, Waltham, MA, USA). Then, formaldehyde agarose gel (1%) electrophoresis was used to assess the integrity of the RNA samples. From each tissue sample, 1 µg of total RNA was reverse transcribed into complementary DNA (cDNA) by RevertAid First Strand cDNA Synthesis Kit (Thermo Fisher Scientific, K1622), following the manufacturer’s instructions.

### 4.3. Oligonucleotide Design and Molecular Cloning of DQA

Three overlapping PCR primers were designed according to the DQA genes available in the GenBank (XM_004782395.2, XM_022510513.1) for *Neovison vison* DQA gene amplification (Table 2). These primers were used in RT-PCR for the amplification of DQA-cDNA fragment. 

Genomic DNA PCR amplification was performed using ExTaq Kit (TaKaRa, RR01AM). The reaction volume of 25 µL comprises of the proviral genomic DNA template, 0.125 μL of Ex Taq, 2.5 μL of Ex Taq Buffer, 2 μL of dNTP mixture, 0.5 μL of both forward and reverse primers (20 μmol/L), and 1 µL cDNA template. The PCR reaction included an initial denaturation cycle of 5 min at 94 °C, followed by 35 cycles of denaturation for 30 s at 94 °C, annealing for 30 s (the optimum annealing temperatures are listed in Table 1), and an extension for 1 min at 72 °C, with a final extension of 10 min at 72 °C. The PCR products were excised from a 1.0% agarose gel, purified using GeneJET Gel Extraction Kit (Thermo Fisher Scientific, K0692), ligated into the pMD19-T vector (TaKaRa, 6013), and transformed into Escherichia coli DH5α competent cells. Then, the plasmid DNA was extracted from the transformed cells using a TIANprep Mini Plasmid Kit (Tiangen, Beijing, China). Three independent clones were sequenced by the Beijing Genomics Institute (Beijing, China).

### 4.4. Multiple Sequence Alignment and Phylogenetic Analysis

Multiple sequence alignment was carried out using the sequence analysis software Lasergene 1 (DNASTAR Inc., Madison, WI, USA) and Clustal X program, version 1.83. A neighbor-joining tree was drawn with MEGA5.0 software (http://www.megasoftware.net), with confidence levels assessed using 1000 bootstrap replications. The sequences of reference DQA genes concluded in the multiple sequence alignment were obtained from GenBank and summarized in Table 3.

### 4.5. Bioinformatics of DQA

The basic physical and chemical properties of mDQA were analyzed using ProtParam online tools (https://web.expasy.org/protparam/). The hydrophobicity of mDQA was predicted using ProtScale online tools (https://web.expasy.org/protscale/). The transmembrane structure of DQA was predicted using TMHMM 2.0 (http://www.cbs.dtu.dk/services/TMHMM-2.0/). The subcellular localization of DQA was predicted using the online software Target P1.1 server (https://www.cbs.dtu.dk//services/TargetP/) and PSORT prediction (https://psor.hgg.jp/form.html). The phosphorylation sites and O and N glycosylation sites of DQA were predicted using the online tools: NetPhos 3.1 server (http://www.cbs.dtu.dk/services/NetPhos/), NetOGlyc 3.1 (http://www.cbs.dtu.dk/services/NetOGlyc-3.1/), and NetNGlyc 1.0 (http://www.cbs.dtu.dk/Services/NetNGlyc/), respectively. The signal peptide of DQA was predicted using online tools SignalP 4.1 (http://www.cbs.dtu.dk/services/SignalP/). The structural-functional areas and functions of DQA were predicted using the online tools Protfun 2.2 (http://www.cbs.dtu.dk/services/ProtFun/). The amino acid sequence of mDQA was subjected to predict the secondary and 3D structures. The secondary structure was predicted using PredictProtein (http://www.predictprotein.org/), while the 3D conformation was predicted using the Swiss-model server (https://www.swissmodel.expasy.org/) for modeling the homologous structure.

## 5. Conclusions

The *DQA* gene of *Neovison vison* was amplified for the first time. The results of this study lay a foundation for further study on the antiviral molecular mechanism of *DQA*. 

## Figures and Tables

**Table 1:** Functional analysis of *DQA* gene-encoding product.

Gene Ontology Category	Odds
Metal-ion-transport	0.039
Growth-factor	0.357
Immune-response	0.129
Stress-response	0.830
Transcription-regulation	0.888
Ion channel	3.174
Voltage-gated-ion channel	12.682
Transporter	0.229
Structural protein	0.107
Hormone	0.154
Receptor	0.041
Signal-transducer	0.958

**Table 2:** Primers and reaction conditions used for PCR amplification of *Neovison vison* DQA gene.

Position	Name	Sequence (5′–3′) bp	Annealing Temperature	Expect Size (bp)
32–665	F1U	TTACTGCTGCTCTGCAACTCA	58	634
	F1L	GTGCTCCACCTTGCAGTCAT		
108–1163	F2U	ATTCTGGGGACTCTTGCCCT	60	1056
	F2L	TGGGAGTTCTGGATCAGGGTT		
1046–1231	F3U	ATTCTGCACCTTGAATGGCTG	60	196
	F3L	CAGTTCTGAGATGAAAGAAAGGAAA		

The positions where the primers bind are in accordance with the published sequence of *Mustela putorius furo* DQA gene (GenBank ID: XM_004782395.2).

**Table 3:** Sequence information used in the construction of phylogenetic trees.

Species	Accession Number
*Mustela pulourius furo*	XM_004782395.2
*Enhydra lutris*	XM_022510513.1
*Ursus maritimus*	XM_008693471.1
*Ailuropoda melanoleuca*	XM_002928248.3
*Odobenus rosmarus divergens*	XM_004417612.1
*Zalophus*	AF502564.1
*Canis lupus*	NM_001011726.1
*Neomonachus schauinslandi*	XM_021684531.1
*Equus caballus*	NM_001142814.1
